# Unsupervised literature mining approaches for extracting relationships pertaining to habitats and reproductive conditions of plant species

**DOI:** 10.3389/frai.2024.1371411

**Published:** 2024-05-23

**Authors:** Roselyn Gabud, Portia Lapitan, Vladimir Mariano, Eduardo Mendoza, Nelson Pampolina, Maria Art Antonette Clariño, Riza Batista-Navarro

**Affiliations:** ^1^Department of Computer Science, College of Engineering, University of the Philippines Diliman, Quezon City, Philippines; ^2^Institute of Computer Science, College of Arts and Sciences, University of the Philippines Los Baños, Laguna, Philippines; ^3^Department of Forest Biological Sciences, College of Forestry and Natural Resources, University of the Philippines Los Baños, Laguna, Philippines; ^4^Young Southeast Asian Leaders Initiative (YSEALI) Academy, Fulbright University Vietnam, Ho Chi Minh City, Vietnam; ^5^Mathematics and Statistics Department, De la Salle University, Manila, Philippines; ^6^Center for Natural Science and Environmental Research, De la Salle University, Manila, Philippines; ^7^Max Planck Institute of Biochemistry, Munich, Germany; ^8^Department of Computer Science, University of Manchester, Manchester, United Kingdom

**Keywords:** relation extraction, information extraction, unsupervised methods, rule-based methods, transformer models, biodiversity

## Abstract

**Introduction:**

Fine-grained, descriptive information on habitats and reproductive conditions of plant species are crucial in forest restoration and rehabilitation efforts. Precise timing of fruit collection and knowledge of species' habitat preferences and reproductive status are necessary especially for tropical plant species that have short-lived recalcitrant seeds, and those that exhibit complex reproductive patterns, e.g., species with supra-annual mass flowering events that may occur in irregular intervals. Understanding plant regeneration in the way of planning for effective reforestation can be aided by providing access to structured information, e.g., in knowledge bases, that spans years if not decades as well as covering a wide range of geographic locations. The content of such a resource can be enriched with literature-derived information on species' time-sensitive reproductive conditions and location-specific habitats.

**Methods:**

We sought to develop unsupervised approaches to extract relationships pertaining to habitats and their locations, and reproductive conditions of plant species and corresponding temporal information. Firstly, we handcrafted rules for a traditional rule-based pattern matching approach. We then developed a relation extraction approach building upon transformer models, i.e., the Text-to-Text Transfer Transformer (T5), casting the relation extraction problem as a question answering and natural language inference task. We then propose a novel unsupervised hybrid approach that combines our rule-based and transformer-based approaches.

**Results:**

Evaluation of our hybrid approach on an annotated corpus of biodiversity-focused documents demonstrated an improvement of up to 15 percentage points in recall and best performance over solely rule-based and transformer-based methods with F1-scores ranging from 89.61 to 96.75% for reproductive condition - temporal expression relations, and ranging from 85.39% to 89.90% for habitat - geographic location relations. Our work shows that even without training models on any domain-specific labeled dataset, we are able to extract relationships between biodiversity concepts from literature with satisfactory performance.

## 1 Introduction

Plants provide food to humans and other terrestrial animals, are habitats to more than 80% of terrestrial species, are the source of clean air and water, and regulate climatic balance (UNDP, 2023). However, there has been a continuous decline in the world's forests and biodiversity (FAO, 2019; FAO and UNEP, 2020). A major contributor to this decline in natural resources is the growing population on Earth, currently around 8 billion, that increases global demand for food and other commodities. Climate change has also curtailed vegetation eventually affecting food production in a number of locations (Lobell et al., 2011; Ray et al., 2019).

In order to ensure that the benefits of land-based ecosystems will be enjoyed for generations to come, the United Nations Sustainable Development Goal (SDG) #15, Life on Land, focuses specifically on managing forests sustainably, halting and reversing land and natural habitat degradation, successfully combating desertification and stopping biodiversity loss (UNDP, 2023). Land restoration and rehabilitation require knowledge of plant species' reproduction and regeneration properties. Precise timing in fruit collection and knowledge of reproductive activities is necessary especially in the tropical regions (Luna-Nieves et al., 2017) where many tree seeds are recalcitrant (Barbedo et al., 2013) and have very short viability, lasting only a few weeks or months under normal conditions (Oshima et al., 2015). Reproductive activities such as flowering and fruiting in the case of plants, are timed events within the species' life cycle that are associated with seasonal timings (Amasino, 2010). As a life event, reproduction is studied in association with its coupling factor, i.e., time (Amasino, 2010; Ehrlén, 2015). Thus, it is impossible to understand reproduction without the context of time. Reproductive patterns may occur in irregular intervals for long periods of time, and may even be affected by habitat variations.

Understanding species' habitat preferences, i.e., the natural home or environment, is another aspect of successful reforestation (Poulin et al., 2013; Lelli et al., 2018; Staples et al., 2020). With the changing ecosystem affected by factors such as climate change, deforestation, and desertification (Cochard, 2001; Gebeyehu and Hirpo, 2019), a complete knowledge of “what grows where,” that traditionally used to be geographic-based distribution of species, is now being augmented by more data-driven approaches that associate information on habitats with distribution data (Morueta-Holme and Svenning, 2018). Hence, information on plant species' reproductive conditions with associated temporal information, and habitats with associated geographic locations will aid in more informed reforestation and cultivation of land.

Sustainable management of land and vegetation must be fueled by long-term and broad-scale understanding of the biology underpinning the reproduction of plant species (Gabud et al., 2019), the foundation of which is biodiversity data available in either structured or unstructured form. There exist widely-used biodiversity databases that contain structured information on species and their occurrences such as the Global Biodiversity Information Facility (GBIF) with about two billion occurrence records (GBIF, 2023), and the Atlas of Living Australia (ALA) containing around 100 million occurrence records (ALA, 2023). Both GBIF and ALA use data standards such as Darwin Core (Wieczorek et al., 2012) to mobilize and deliver biodiversity data. Darwin Core is the internationally agreed data standard that includes a glossary of terms, i.e., fields or attributes, intended to facilitate the sharing of information on biodiversity by providing identifiers, labels, and definitions (TDWG, 2023). Darwin Core is primarily based on taxa, their occurrence, and related information which is reflected in the types of data stored in GBIF and ALA, e.g., species' occurrence data. Darwin Core includes terms that are used to represent information on species' reproductive condition and habitats, namely, Reproductive Condition and Habitat; they can, for example, be populated with values such as “*in bloom”* and “*oak savanna,”* respectively. While both GBIF and ALA publish data on species that include these two fields, a substantial number of their occurrence records have these fields blank and unpopulated. For example, in GBIF, the 61,392 occurrence records for *Dipterocarpaceae* (a family of tropical rainforest trees) provide information on reproductive condition and habitat for only 37,624 and 4,332 records, respectively. Meanwhile, in ALA, out of the 1,248 occurrences of a similar family, *Dipterocarpaceae Blume*, only 419 and 870 provide reproductive condition and habitat information, respectively.

Although there is a growing movement to make research data more reusable and accessible in structured form, scientific literature still remains a major repository for much of our knowledge about the natural world and represents centuries of investment (Thessen et al., 2012; Le Guillarme and Thuiller, 2022). This is because the research literature often contains detailed descriptions of the data collection methods and analytical procedures used, which can be essential for understanding and interpreting findings. For instance, detailed information on species reproduction and habitat is narrated well in literature, e.g., “*In 1981, flowering was heavy in Kepong and Pasoh, but weak in Gombak and Ampang”* and “*The main observation site was conserved forest at Dongmakhai.”* Currently, over 60 million pages of legacy biology text are scanned and made available online through the Biodiversity Heritage Library (BHL, 2023) and thousands of new digital pages are published every month in open-access biology and ecology journals (Cornford et al., 2021). This represents an enormous amount of unstructured information that can potentially be exploited in data-driven studies. If tools are developed to automatically extract fine-grained information from these sources and feed such information into open biodiversity databases in a structured form, then this information will be much more accessible and more useful for large-scale studies (Le Guillarme and Thuiller, 2022). This could potentially allow researchers to better understand the relationships between species and their habitats, and to develop more effective conservation strategies.

Information extraction (IE) is an umbrella term for tasks that seek to automatically extract structured information from unstructured text. With the exponential growth of digitized literature over the years, IE has become increasingly pertinent, due to its role in (semi-)automatically populating databases with content (Ravikumar et al., 2015; Lee et al., 2018; Paragkamian et al., 2022). Relation extraction (RE) is an IE task that is concerned with the identification of semantic relationships between entities or concepts in text. This task predicts whether a relationship holds between two entities (or concepts), based on the context of the sentence. For example, in the sentence “*The flowering commenced in July and continued until October 2001, with viable seed fall occurring from early December 2001 until early February 2002,”* a relation extraction system should be able to identify the relationship between the reproductive condition mention “*flowering”* and the temporal expression “*October 2001,”* but no relation between “*flowering”* and “*December 2001.”* This information can prove to be crucial in analyzing the reproductive behavior of a species of interest. Harvesting these details from literature will enable big data-centric discovery focused on understanding plant species' reproductive patterns and habitats.

In this paper, we focus on extracting the following, with a view to enabling the curation of biodiversity databases: (1) relationships between expressions denoting a plant species' reproductive condition and the time or duration when those conditions hold, and (2) relationships between habitats and their geographic locations. Our work will thus ultimately support the analyses required for planning efforts in plant regeneration, and land restoration and rehabilitation. The main contributions of our work include: (1) the creation of a new RE corpus—drawn from the biodiversity-focused corpus presented in the work of Gabud et al. (2019)—in which the above-mentioned relationship types were manually labeled, thus providing a new gold standard dataset; and (2) a comparative evaluation and combination of two types of unsupervised approaches: rule-based and transformer-based methods for RE.

In the remainder of this paper, we first provide a review of prior work related to our study (Section 2). This is followed by a formalization of the problem we are aiming to solve (Section 3) and a description of the dataset we developed and used in our experiments (Section 4). Importantly, we present details of the various unsupervised RE approaches that we developed (Section 5), and the results of evaluating and combining them into one hybrid model (Section 6). We then analyze our results, discuss their implications and limitations (Section 7) before providing a summary of our findings and directions for future work (Section 8).

## 2 Related work

In this section, we provide a review of previously reported work on natural language processing and information extraction more specifically, that have been applied to the biodiversity domain and are considered to be relevant to our own work.

### 2.1 Text mining and natural language processing

The growing volume of scientific literature on biodiversity has led to a focus on the development of computational methods for extracting meaningful information from unstructured textual data (Farrell et al., 2022; Paragkamian et al., 2022). This computational task is known as text mining, and it has been used to identify trends, patterns, and relationships that would otherwise be difficult to detect. Text mining has successfully been applied to biodiversity literature (Batista-Navarro et al., 2016, 2017; Gabud et al., 2017; Parr and Thessen, 2018; Chaix et al., 2019; Lee et al., 2019; Page, 2019). There has been significant progress in the development of natural language processing (NLP) and machine learning (ML) algorithms that can be used to automatically annotate taxonomic text (Lücking et al., 2022), identify taxonomic names in text (Le Guillarme and Thuiller, 2022), link person names to biodiversity data (Groom et al., 2020), and extract phenotypic traits from text (Thessen et al., 2018). However, more tools and services are needed to scale up the accessibility of biodiversity data (Thessen et al., 2022).

### 2.2 Information extraction

Information extraction (IE) is a specific task within NLP that deals with the automatic process of extracting structured information from unstructured data sources such as scientific publications and books. One IE subtask that has gained much attention is Named Entity Recognition (NER), i.e., the task of identifying named entities such as taxonomic names. Most work on NER in the biodiversity domain have been focused on the extraction of taxonomic names. Early work on taxonomic NER systems were rule-based (Koning et al., 2005; Sautter et al., 2006) and made use of handcrafted rules based on regularities in taxon naming conventions to recognize mentions of taxa in text. Researchers also proposed dictionary-based approaches that match text against a predefined dictionary of species names (Leary et al., 2007; Gerner et al., 2010; Pafilis et al., 2013). However, taxonomic NER systems that were developed more recently employ either a deep neural network-based approach (Le Guillarme and Thuiller, 2022) or a hybrid approach combining ML with rules and dictionaries (Mozzherin, 2022; Thessen et al., 2022).

NER methods that consider other types of entities include the ML-based models developed by Ahmed et al. (2019) and Nguyen et al. (2019) for recognizing taxon names, person names, locations and temporal expressions in biodiversity literature. More recently, Mora-Cross et al. (2022) employed ML to extract plant phenological data from specimen labels, which is a textual document that follows a specific format. In our work, we assume that recognized named entities are provided as part of the input to our RE methods. While the development of biodiversity NER methods is outside of the scope of this paper, it is important to note that pre-identified mentions of reproductive conditions, time, habitats, and locations are required for RE.

### 2.3 Relation extraction

Relation extraction (RE) is another IE task that involves detecting pre-defined semantic relationships between entities mentioned in text (Song et al., 2022). There are a few available methods for relation extraction in the biodiversity domain. The work by Chaix et al. (2019) focused on extracting *lives_in* relationships between bacteria species and the location where it lives, which can either be a habitat or a geographic entity. Nguyen et al. (2019), due to lack of gold standard annotated relations, employed an unsupervised pattern-based method to identify binary relations that pertain to the occurrence of species in specific geographic locations, habitats, or points of time. Kopperud et al. (2022) extracted related taxonomic names and geographic locations by training an ML-based relation classifier to determine if a given sentence explicitly stated or strongly implied that a given taxon was found in the mentioned location, or not.

RE approaches can be categorized into three: early rule-based, supervised, and zero-shot learning approaches. Early methods for extracting relations between entities were based on rule templates that were created by experts (Fundel et al., 2007; Zhang et al., 2009; Nguyen et al., 2015). These rules were designed to capture the syntactic patterns that are associated with different types of relations, as observed in a corpus. Rules have the advantage of being highly interpretable, as they can be easily understood by humans. However, rule-based methods have two main limitations: they can be time-consuming to create and they are domain-dependent. To alleviate the burden on human experts, methods to automatically construct rules were developed (Carlson et al., 2010; Zheng et al., 2019).

Supervised methods for RE have been extensively studied (Yan et al., 2021). Early work on superpervised methods trained an ML-based model on a manually labeled dataset, and then employed the model to classify the relation. Models can be feature-based (Miller et al., 2000; Kambhatla, 2004) or kernel-based (Zelenko et al., 2002; Culotta and Sorensen, 2004). The more advanced Deep Neural Network (DNN) methods have been shown to outperform traditional supervised methods for RE. These DNN models learn higher-order, abstract feature representations from sentences. With the emergence of DNNs, models that employ neural architectures such as convolutional neural networks (CNNs; Liu et al., 2013; dos Santos et al., 2015), recurrent neural networks (RNN; Zhang and Wang, 2015; Vu et al., 2016), graph convolutional networks (GCN; Zhu et al., 2019), attention-based neural networks (Wang et al., 2016; Xiao and Liu, 2016), and transformer-based language models (Vaswani et al., 2017; Lee et al., 2022) have been utilized for RE tasks. Like traditional ML-based models, DNN-based models learn features from data. This gives them strong generalization ability, adaptability, and scalability. However, training or fine-tuning them for downstream applications such as RE requires labeled data (Zhao et al., 2023). Since we have limited labeled data, supervised methods such as those mentioned above cannot be applied in our study.

In recent years, approaches to IE that are considered to be *zero-shot*, i.e., requiring no labeled data, have started gaining momentum (Liu et al., 2020; Cheng et al., 2021; Du and Cardie, 2021; Li et al., 2022); these leverage models that were originally trained for other tasks, e.g., machine reading comprehension (MRC), question answering (QA). For instance, Levy et al. (2017) reduced RE to the problem of answering simple reading comprehension questions. They mapped each relation type *R(x, y)* to at least one parameterized natural-language question *q*_*x*_ whose answer is *y*. For example, the relation *educated_at(x, y)* can be mapped to “*Where did x study?”* and “*Which university did x graduate from?”* The success of these types of RE methods is primarily due to the significant developments in and availability of transformer-based pre-trained language models (PLMs; Devlin et al., 2019; Liu et al., 2019; Raffel et al., 2020). These are models that were pre-trained on large-scale corpora using unsupervised learning objectives such as masked language modeling. These PLMs can be fine-tuned for downstream tasks, such as question answering (QA) and natural language inference (NLI), using relatively smaller amounts of task- or domain-specific labeled data. Zero-shot methods have some advantages over traditional RE methods, including: (a) the ability to generalize and extract previously unseen relations; and (b) significant reduction in the labeling cost associated with RE because only a small amount of labeled data is required, i.e., test samples for evaluating the model. However, as the models underpinning zero-shot methods were trained on out-of-domain data, they might struggle to perform well on RE for specialized domains (such as biodiversity). One way to alleviate this issue is by constructing a hybrid model that combines the strengths of different types of approaches, e.g., zero-shot and rule-based approaches. Our work thus explores the development and evaluation of such a hybrid approach to RE.

### 2.4 Annotated corpora

Text collections that include manually provided annotations (also known as gold standard corpora) are valuable resources for NLP research as they support the development and evaluation of various methods. In recent years, gold standard corpora drawn from the biodiversity domain have increasingly become more available.

The first proposed biodiversity corpora contain taxonomic name annotations to support taxonomic NER. These are Linnaeus-100 (Gerner et al., 2010) that consists of 100 randomly selected full-text documents from PubMedCentral (PMC), and Species-800 (Pafilis et al., 2013) that is based on 800 PubMed abstracts. The latter is a collection of 100 abstracts from each of the following eight subject areas: bacteriology, botany, entomology, medicine, mycology, protistology, virology, and zoology, and thus contains annotations that represent many taxonomic groups.

Meanwhile, biodiversity corpora that were introduced more recently cover multiple types of entities pertaining to information on species occurrence reported in biodiversity literature. BIOfid (Ahmed et al., 2019) is a collection of German documents drawn from historical scientific literature on the biodiversity of plants, birds, moth and butterflies, that were converted to plain text by optical character recognition (OCR). It includes annotations of taxon names, locations, temporal expressions, person names, and organization names. Similarly, documents in the COPIOUS corpus (Nguyen et al., 2019) were annotated to capture taxon names, locations, temporal expressions, and person names, as well as mentions of habitats. Thus far, COPIOUS is the largest biodiversity corpus that consists of 668 documents downloaded from the Biodiversity Heritage Library (BHL) with over 26K sentences and more than 28K annotated entities. Parallel to the development of COPIOUS, Gabud et al. (2019) developed DipteroMine, a biodiversity corpus that contains additional annotations pertaining to reproductive information, i.e., the reproductive condition of species. It consists of abstract-length documents: 250 from BHL, 150 from journal articles, and 100 from government reports. The most recent addition to the list of biodiversity corpora is BiodivNERE (Abdelmageed et al., 2022). Drawn from biodiversity dataset metadata and abstracts, it comes with two datasets, one supporting NER and the other supporting Relation Extraction (RE). In terms of named entity annotations, it covers six entity types, i.e., organism, environment, quality, location, phenomena, and matter. The RE dataset, meanwhile, is based on a subset of the NER dataset and includes annotations of binary relationships between different entity types, such as *occur_in* (between organism and environment), *influence* (between organism and process) and *have/of* (between quality and environment).

This work makes use of the journal articles included in the DipteroMine corpus (Gabud et al., 2019), a gold standard corpus for biodiversity NER that was designed in accordance with the annotation scheme used in the COPIOUS project (Nguyen et al., 2019). In addition to taxonomic names, geographic locations, and temporal expressions, the DipteroMine corpus contains manual annotations pertaining to habitat and reproductive condition mentions which are relevant to our study. Table 1 shows a comparison of existing corpora containing manually annotated biodiversity-relevant named entities and relations.

**Table 1:** Characteristics of existing corpora with manual annotations of biodiversity-related named entities and relations.

**Corpus**	**Doc type**	**Docs**	**Sent**	**Words**	**NE type (count)**	**RE type (count)**
Linnaeus	PMC full paper	100	17,580	502,507	Taxon (4,259)	NA
S800	PubMed abstract	800	8,064	201,981	Taxon (3,708)	NA
BIOfid	German historical literature	969	15,833	Undisclosed	Taxon (15,085) Loc (6,785) Time (5,197) Person (5,393) Org (1,085) Other (7,849)	NA
COPIOUS	BHL pages	668	26,277	298,230	Taxon (12,227) Geo Loc (9,921) Temp Exp (2,889) Habitat (2,210) Person (1,554)	NA
BiodivNERE	Dataset metadata, PubMed abstract	150	2,398	102,113	Organism (2,602) Loc (310) Env (1,666) Matter (1,053) Phenomena (724) Quality (3,627)	Org-Env (392) Org-Mat (189) Org-Qua (865) Env-Env (6) Env-Mat (267) Env-Phe (430) Env-Loc (46) Env-Qua (744) Phe-Phe (4) Phe-Loc (27) Phe-Qua (300) Other (730)
DipteroMine	Online journal	151	5,045	99,359	Taxon (1,460) Geo Loc (711) Temp Exp (787) Habitat (475) Person (36) Rep Cond (539) Hab Att (115) Hab AttVal (126)	Rep-Tem (1,404) Hab-Loc (413)

“NA” stands for “not applicable.”

## 3 Problem formulation

We now provide a formal definition of our relation extraction task. Given an input sentence *I* that is a sequence of tokens [*t*_0_, *t*_1_, ..., *t*_*n*_], a source entity *E*_*S*_ = [*t*_*i*_, ..., *t*_*j*_] and a target entity *E*_*T*_ = [*t*_*u*_, ...., *t*_*v*_], we treat the RE task as a binary classification task, whereby the input is the triple (*I, E*_*S*_, *E*_*T*_), and the output is *y*∈{0, 1} where 1 indicates that a relationship from the source entity to the target entity (*E*_*S*_→*E*_*T*_) exists, otherwise 0. In this work, we focus on the two relation types described below.

- **has_time relation**: This relation holds between a reproductive condition mention and a temporal expression, i.e., “*reproductive condition* has_time *temporal expression*,” whereby the reproductive condition mention is considered to be the source and the temporal expression serves as the target. In this type of relation, the temporal expression provides additional information on the reproductive condition.
- **has_location relation**: This holds between a habitat mention and a geographic location, i.e., “*habitat* has_location *geographic location*,” whereby the habitat mention is considered to be the source and the geographic location is the target.

## 4 Dataset

In this work, we utilized documents that were drawn from the DipteroMine NER gold standard corpus proposed by Gabud et al. (2019), which took inspiration from the design of the COPIOUS dataset (Nguyen et al., 2019). The DipteroMine corpus was developed to support the extraction of information on the distribution and reproductive patterns of forest tree species belonging to the *Dipterocarpaceae* family, more commonly known as dipterocarps. It consists of one- to two-paragraph documents that were manually selected from online environmental science and ecology journal repositories, e.g., Journal of Tropical Ecology, Journal of Ecology, Journal of Biosciences, and Forest Ecology and Management. These scholarly articles on dipterocarps were retrieved using keywords such as “*flowering,” “fruiting,” ‘mass flowering,” “phenology,”* and “*masting.”* Based on this process, a total of 151 abstract-length documents were included in the said corpus.

In our RE work, we are particularly interested in capturing relationships between the following types of entities: habitat, geographic location, temporal expression, and importantly, reproductive condition. The lattermost type includes expressions that pertain to the reproductive status of dipterocarps, or seasonal events involving them, e.g., “*sterility,” “budburst,”* and “*flowering.”* Table 2 provides descriptions and examples for each of our entity types of interest. From the DipteroMine corpus, we selected sentences that contain at least one entity pair, i.e., either a pair of habitat and geographic location mentions, or a pair of reproductive condition and temporal expression mentions. We then produced relation annotations by creating instances, each of which is in the form (*I, E*_*S*_, *E*_*T*_, *y*), where *I* is the input sentence, *E*_*S*_ is the source entity, *E*_*T*_ is the target entity, and *y* is the relation label which is set to 1 if a binary relation between the source and target entities hold, otherwise *y* is set to 0. As mentioned in the previous section, we decided to focus on two types of relations. One relation type is the has_time relation which holds between a reproductive condition mention (*E*_*S*_) and a temporal expression (*E*_*T*_). The other type is the has_location relation which holds between a habitat mention (*E*_*S*_) and a geographic location (*E*_*T*_). Our dataset contains all occurrences of the *E*_*S*_ and *E*_*T*_ pairs found in every sentence in the corpus. For instance, if a sentence contains one habitat mention and two geographic location mentions, two has_location pairs are generated as relation instances, as exemplified in Table 3.

**Table 2:** Descriptions and examples of our biodiversity concept types of interest.

**Concept**	**Description**	**Example**
Habitat	Environments in which organisms live.	“*In the [lowland mixed dipterocarp forests] of Borneo the Dipterocarpaceae can comprise roughly 107 of species”*
Geographic Location	Any identifiable point or area in the planet, ranging from continents, major bodies of water, named landforms, countries, states, cities, and towns.	“*The main obervation site was conserved forest at [Dongmakhai] ([18deg20'03”N, 102deg30'5”E], 190m a.s.l.)”*
Reproductive Condition	Indicators of the specimens' reproductive behavior.	“*There were two [flowerings] in March to May, and one in August during this period.”*
Temporal Expression	Spans of text pertaining to points in time.	“*Most fruit fall occurred from the [end of July] to [mid-August].”*

**Table 3:** Sample has_location relation data instances for a sentence (*I, E*_*S*_, *E*_*T*_, *y*).

	**Sentence (*I*)**	**Habitat (*E*_*S*_)**	**Geo. Location (*E*_*T*_)**	***y***
1	“*The main observation site was conserved forest at Dongmakhai (18deg20'03”N, 102deg30'5”E, 190m a.s.l.).”*	conserved forest	Dongmakhai	1
2	“*The main observation site was conserved forest at Dongmakhai (18deg20'03”N, 102deg30'5”E, 190m a.s.l.).”*	conserved forest	18deg20'03”N, 102deg30'5”E	1

Source of sentence: Kato et al. (2008).

Two annotators manually provided the label *y* for each data instance (*I, E*_*S*_, *E*_*T*_, *y*). Based on the sentence input *I*, *y* is set to 1 if the two entities *E*_*S*_ and *E*_*T*_ have a relationship, and set to 0 otherwise. One annotator is a Biology degree holder, while the other annotator is a Computer Science student. They worked on the annotation task independently. We then randomly split the set of annotated instances into a training set (70%), development set (10%), and test set (20%).

## 5 Methods

In this section, we present our methods for extracting (1) related reproductive condition and temporal expressions (i.e., has_time relations), and (2) related habitat and geographic location mentions (i.e., has_location relations).

### 5.1 Rule-based approaches

We designed two traditional rule-based approaches for RE. We based our rules on syntactic patterns observed in the sentences in our training corpus. The first one extracts relations based on the distance of the dependency between a given pair of entities, and the second one implements pattern-matching using regular expressions.

#### 5.1.1 Dependency distance

**Figure 1:**
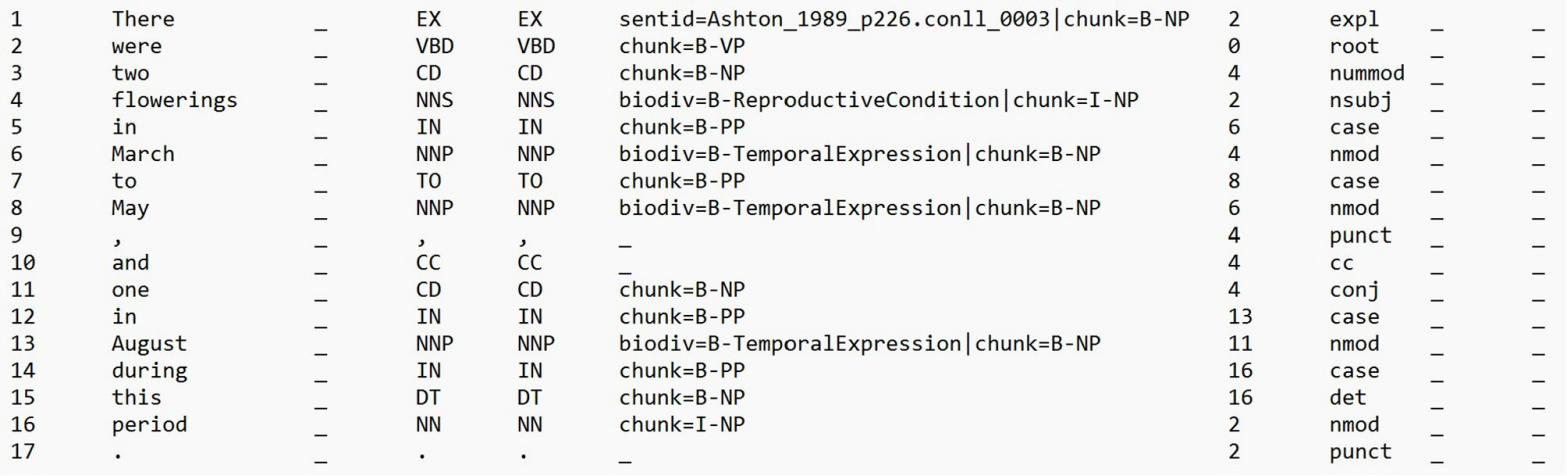
An excerpt from an example CoNLL-U file that was used as input for Grew. Source of sentence: Ashton (1989).

Dependency distance refers to the number of edges traversed between the head and dependent words along the shortest path in the dependency parse tree of a sentence. In a dependency parse tree, each word or token in a sentence is represented as a node, and the syntactic relationships between words are represented as directed edges. In this work, we used the dependency distance between two entity spans, *E*_*S*_ and *E*_*T*_, that are contained in an input sentence *I*, as basis for RE. We used the Stanford Dependency Parser^1^ to generate the dependency parse tree of each of the sentences in our dataset. Given an input sentence *I*, that is a sequence of tokens [*t*_0_, *t*_1_, ..., *t*_*n*_], the parser returns a sentence's dependency parse tree in the CoNLL-U format,^2^ a standard means for storing dependency and feature structures of sentences. We implemented a pre-processing step to make the tab-separated value (TSV) CoNLL-U file include additional columns corresponding to the manual annotations for named entities, i.e., mentions of habitats, geographic locations, reproductive conditions, and temporal expressions. If a token *t*_*i*_ in an input sentence *I* belongs to a named entity, we add the entity's type to the token's FEATS field, i.e., the sixth column in the CoNLL-U file that contains a list of morphological features. Specifically, we added an additional feature called *biodiv*, and set its value to the named entity type following the BIO (Beginning, Inside, Outside) sequence labeling format, e.g., *biodiv=B-Habitat* for the first token of a habitat entity. Figure 1 shows an excerpt from an example processed CoNLL-U file. This file was then used as input to *Grew*,^3^ a graph rewriting system for manipulating linguistic representations (Bonfante et al., 2018; Guillaume, 2021). We selected this tool as it readily supports pattern matching over linguistic representations (including those written in the CoNLL-U format). Furthermore, it is well-documented and is continuously being maintained.

*Grew* comes with a component called *Grew-match* that executes queries over documents in a given corpus, whereby a query is in the form of a pattern and all items matching the pattern are returned. An input pattern describes the nodes (node clauses) and relations (edge clauses) that must be found in the text of the documents. A *node clause* is a node described by an identifier and some constraints in its feature structure; an example of a node clause is *bdREP [biodiv=B-ReproductiveCondition, upos=NN]*, where *bdREP* is the identifier, and *biodiv=B-Habitat, upos=NN* are the constraints that should be met. Meanwhile, an *edge clause* specifies the existence of an edge between two nodes with or without additional constraints. For example, *bdREP*→*bdTEMP* describes an edge from the node with identifier *bdREP* to the one with identifier *bdTEMP* without any additional constraint, while *bdREP -[nsubj|obj]*→*bdTEMP* specifies that the edge label (i.e., dependency type) should be either *nsubj* (nominal subject) or *obj* (direct object). We supplied patterns to *Grew-match* in order to extract related entities whose dependency distance is an integer *n*. To determine the best value for *n*, we performed experiments on our development set, as described in Section 6.2.1.

**Figure 2:**
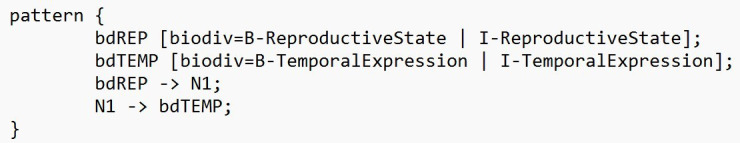
Example *Grew-match* pattern with a dependency distance of 2.

**Figure 3:**
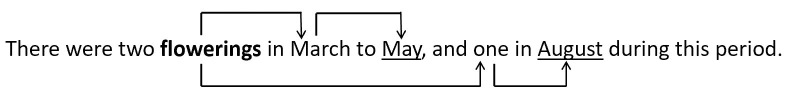
Related reproductive condition (in bold) and temporal expression (underlined) extracted using a *Grew-match* pattern with maximum dependency distance *n* = 2.

We handcrafted *Grew-match* patterns that made use of nodes that are biodiversity named entities and edges without additional constraints, to extract related entities. An example of a pattern that extracts entities with a dependency distance of 2 is shown on Figure 2. Together with an input sentence *I* containing a feature structure in CoNLL-U format, our created patterns, when fed into *Grew-match*, were able to analyze sentences and extract entities separated by a dependency distance of *n*. Using this method, we are able to associate habitats with their geographic locations (has_location relation), and determine a species' reproductive condition at a specific point in time (has_time relation). Figure 3 presents the has_time relations extracted from an example sentence, using the pattern (with dependency distance *n* = 2) shown in Figure 2.

#### 5.1.2 Regular expression-based rules

Apart from handcrafting patterns based on dependency distances, we also created rules to extract related biodiversity entities by observing syntactic patterns, i.e., word order, in the sentences they appear in. These patterns were then captured by a set of regular expressions (regexes). Given an input sentence *I* that is a sequence of tokens [*t*_0_, *t*_1_, ..., *t*_*n*_], we firstly categorized every token *t*_*i*_ according to the following types: *source, target, delimiter*, and *other* as shown in Table 4. We define *source* as a token that belongs to a named entity identified as a source entity type, i.e., either reproductive condition (for has_time relations) or habitat (for has_location relations). Meanwhile, *target* is a token that belongs to a named entity considered to be a target entity type, i.e., temporal expression (for has_time relations) or geographic location (for has_location relations). *Delimiter* is a token that acts as a separator in an enumeration, i.e., a comma or semicolon. Any token that is neither a part of a named entity nor a delimiter is categorized as *other*. We convert each token *t*_*i*_ into a character representation of the token's type. Hence, we convert a sentence into a string of characters, wherein each character is either *S* (source), *T* (target), *d* (delimeter), or *o* (other). We use this sequence of token types as input to our regex method implemented using Python's regular expression module, *re*.

**Table 4:** Types of tokens we designed for the regular expression-based rules.

**Token type**	**Symbol**	**Description**	**Entity type**
Source	S	A token that belongs to a named entity category identified as a source category	Reproductive condition or habitat
Target	T	A token that belongs to a named entity category identified as a target category	Temporal expression or geographic location
Delimiter	d	A token that is a separator in an enumeration	Comma or semicolon
Other	o	Any token that is neither a part of a named entity nor a delimiter	

To extract relations, we created the following regex rules:

1. [S]+(o)?(To|Td|T)+—*source* token that may or may not be followed by one *other* token, then followed by one or more *target* tokens that may or may not be delimited by any token, and
2. (? < !S)(To|Td|T)*T(o)?[S]+—one or more *target* tokens that may or may not be delimited by any token that is not immediately preceded by a *source* token, and followed by a *source* token that may or may not be preceded by one *other* token.

**Figure 4:**
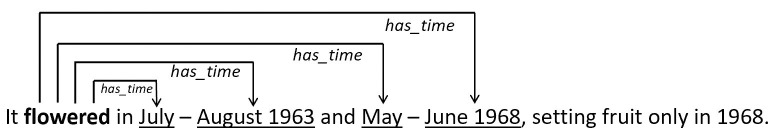
Example sentence with entity pairs that matched the rule [S]+(o)?(To|Td|T)+, where S corresponds to reproductive condition (in bold) which is the *source* entity, T corresponds to temporal expression (underlined) which is the *target* entity and o refers to *other* tokens. Source of example sentence: Medway (1972).

The entity spans (i.e., source and target tokens) that match the patterns above are perceived to be related, and are given the value 1 for *y*. We formulated the two regular expressions above to extract related consecutive entities in a sentence. Figure 4 shows a sample sentence with a text span that matches regex rule 1 above.

### 5.2 Transformer-based approaches

As mentioned in Section 2.3, the emergence of transformer-based models (Vaswani et al., 2017) has allowed researchers to cast RE as a natural language understanding problem such as question answering or machine reading comprehension. One of the key features of transformer-based models is its transfer learning capability, whereby they learn rich representations of natural language via pre-training on large-scale corpora using unsupervised learning objectives such as masked language modeling. The pre-trained models can then be fine-tuned on specific downstream tasks (e.g., question answering) using relatively smaller amounts of task-specific labeled data. Fine-tuning a pre-trained transformer often leads to better performance, faster convergence, and improved generalization compared to training models from scratch.

In this work, we cast our RE problem as: (1) a boolean question answering (boolean QA) task, and (2) a natural language inference (NLI) task, both employing transformer-based language models. Given an input sentence *I* and two entities *E*_*S*_ and *E*_*T*_ for which we wish to determine whether a relation holds, we systematically generate a passage-question pair or a premise-hypothesis pair, which serves as input to the boolean QA and NLI models, respectively. The two entities are considered only if one of them is a reproductive condition mention (*E*_*S*_) and the other is a temporal expression (*E*_*T*_), or if one of them is a habitat mention (*E*_*S*_) and the other is a geographic location (*E*_*T*_). This, respectively, means that we are aiming to determine if a has_time or has_location relation possibly holds between them.

#### 5.2.1 Boolean question answering

We determine the existence of a has_time relationship between a reproductive condition and a temporal expression, and a has_location relationship between a habitat and a geographic location using transformer-based models that were fine-tuned on the BoolQ dataset (Clark et al., 2019). BoolQ is a dataset that consists of *Yes*/*No* questions; it comes with 15,942 examples that are naturally occurring, i.e., generated in unprompted and unconstrained settings. Each example in BoolQ is a triple of the form *(question, passage, answer)*, where question is the *Yes*/*No* question, passage is the context for answering the question, and answer is either *Yes* or *No*. For our boolean QA task, we used two transformer models that were fine-tuned on BoolQ and are available in HuggingFace,^4^ specifically, one based on RoBERTa (roberta-base-boolq)^5^ and the other based on T5 (t5-base-finetuned-boolq).^6^

RoBERTa was introduced by Liu et al. (2019) and was built upon the original BERT model (Devlin et al., 2019) that was released by Google in 2018. Specifically, it introduced optimized key hyperparameters and was trained with much larger mini-batches. The roberta-base-boolq model that was fine-turned for boolean QA accepts a question and a passage as input, and returns probabilities for the *Yes* and *No* answers (i.e., the possible labels) as output.

Text-to-Text Transfer Transformer (T5) is an encoder-decoder model pre-trained on a mixture of unsupervised and supervised tasks; each task was converted into a sequence-to-sequence format, where each of the input and the output is a sequence of tokens (Raffel et al., 2020). T5 distinguishes between different NLP tasks by requiring an indicative prefix to be prepended to the input sequence. For example, T5, already fine-tuned for the machine translation and question answering tasks, interprets the prefixes “*translate English to German:”* and “*question: ... context: ...”* to mean that, respectively, the supplied input should be translated to German, and that the input is a question that needs to be answered based on the supplied passage (i.e., the context). The t5-base-finetuned-boolq model, fine-tuned on the BoolQ dataset, accepts as input a question and a passage with the “*question: ... context: ...”* prefix, and returns either *Yes* or *No* (as a sequence).

Given a data instance (*I, E*_*S*_, *E*_*T*_, *y*), the input sentence *I* is taken as the passage, while the question is created by populating either of the following question templates with *E*_*S*_ and *E*_*T*_, depending on the types of the two entities:

- *Is there*<*habitat*> *in*<*geographic location*>*?*
- *Did*<*reproductive condition*> *event happen on*<*temporal expression*>*?*

Our question templates for the boolean QA models and examples with their corresponding outputs are shown in Table 5. We say that a relationship between two entities exists if the model's predicted class is *Yes*, otherwise the entities are considered to be unrelated.

**Table 5:** Examples of populated question templates for generating inputs (passages and corresponding questions) for the boolean QA model, together with the corresponding expected outputs.

**Relation type**	**Question template**	**Examples**		
		**Passage/context**	**Question**	**Answer**
has_location	Is there < **Habitat**> in < **Geographic Location**>?	*Bukit Sai and Lesong belong to the lowland dipterocarp forest types with D. aromatica being the predominant species*.	Is there **lowland dipterocarp forest** in **Bukit Sai**?	Yes
has_time	Did < **Reproductive Condition**> event happen on < **Temporal Expression**>?	*It flowered in July - August 1963 and May - June 1968, setting fruit only in 1968*.	Did **fruit** event happen on **August 1963**?	No

A relation holds between two given entities (in bold) only if the boolean QA model predicts *Yes* as the output label. Source of input sentences (passage): Medway (1972) and Lee (2000).

#### 5.2.2 Natural Language Inference

We also cast our RE problem as a natural language inference (NLI) problem that we also addressed using a transformer-based model. NLI is the task of determining whether a *hypothesis* is true (entailment), false (contradiction), or unverifiable (neutral) given a *premise* which corresponds to some known knowledge about the subject. We selected T5 as our model, considering that NLI is one of the downstream NLP tasks for which T5 was already fine-tuned (Raffel et al., 2020). Specifically, we used the T5-large (t5-large) model^7^ with 770 million parameters. Similar to our boolean QA methods, we systematically generated a premise-hypothesis pair which serves as input to the NLI model. Here, the input sentence *I* is taken as the premise, while the hypothesis is created by populating either of the following sentence templates with *E*_*S*_ and *E*_*T*_:

- *The*<*habitat*> *was in*<*geographic location*>.
- *The*<*reproductive condition*> *event happened on*<*temporal expression*>.

Table 6 provides some example inputs for the T5-based NLI model, and its expected outputs. For our purposes, a relationship between two entities exists only if the model's predicted class is entailment; otherwise the entities are considered to be unrelated.

**Table 6:** Examples of populated hypothesis templates for generating inputs (premise-hypothesis pairs) for the NLI model, together with the corresponding expected outputs by the NLI model.

**Relation type**	**Hypothesis template**	**Examples**		
		**Premise**	**Hypothesis**	**Output**
has_location	The < **Habitat**> was in < **Geographic Location**>.	*Bukit Sai and Lesong belong to the lowland dipterocarp forest types with D. aromatica being the predominant species*.	The **lowland dipterocarp forest** was in **Bukit Sai**.	entail-ment
has_time	The < **Reproductive Condition**> event happened on < **Temporal Expression**>.	*It flowered in July - August 1963 and May - June 1968, setting fruit only in 1968*.	The **fruit** event happened on **August 1963**.	contra-diction

A relation holds between two given entities (in bold) only if the NLI model predicts entailment as the output label.

Due to variations in noun forms or verb tenses, the automatically generated hypothesis (for NLI) and question (for boolean QA) may not necessarily be grammatically correct; for instance, the example for the has_time relation in Table 6 would be more correct if it reads “*The fruiting event happened on August 1963.”* Nevertheless, we did not carry out any engineering on our templates to handle such variations for both boolean QA and NLI, as we expected the transformers-based models to be robust to such grammatical errors.

### 5.3 Hybrid approach: rules and transformers

In order to improve performance and reduce the required computational resources, we designed a two-step solution to our RE problem. Here, we combined our rule-based syntactic pattern matching and transformer-based approaches. The first step is to extract relations using our regex rules. These are the regular expressions we designed to extract consecutive entities in a sentence. It is worth noting that between our two rule-based methods, the regex-based one was chosen over the one based on dependency distance, as the former does not require the optimization of any parameters, unlike the latter which relies on the careful selection of the value of *n*, the maximum dependency distance.

The instances that were identified as not pertaining to any relations using the first step, are fed into the second step. In this step, our transformer-based model is applied on the remaining instances. This step produces a set of related entities using less computational resources compared to running the transformer-based model on the entire dataset.

We investigated the incorporation of an enhancement to our hybrid approach: the use of compound entities in filling in the hypothesis templates instead of using single entity mentions, where applicable. We designed rules to identify multiple, consecutive entities in a given sentence that belong to the same entity type and thus comprise a compound entity *E*_*comp*_. The regular expression that was designed to extract *E*_*comp*_ is (*Et*|*E*){2, }, where *E* is a named entity of a specific type, and *t* is any token. *E*_*comp*_ consists of consecutive entities belonging to the same entity type *E*, which may or may not be delimited by a token (*t*). For example, given the sentence “*It flowered in July - August 1963 and May—June 1968, setting fruit only on 1968,”* the reproductive condition is expressed by the mention “*flowered”* and the compound temporal expression is “*July—August 1963 and May—June 1968.”* Instead of populating a hypothesis template for every temporal expression, we formulated only one hypothesis: “*The flowered event happened on July—August 1963 and May—June 1968.”*

## 6 Evaluation and results

In this section, we assess the reliability of our RE corpus and present the results of the experiments we performed to evaluate the performance of each of the RE approaches presented in Section 5.

### 6.1 Reliability of RE annotations

Our corpus consists of scholarly articles that contain manual annotations of entities of type geographic location, habitat, and temporal expression. Additionally, it also contains annotations of spans of text pertaining to the reproductive condition of species. It is a subset of the corpus presented in Gabud et al. (2019). To facilitate the annotation of relations between entity pairs, we automatically identified (1) co-occurring mentions of reproductive condition and temporal expressions, and (2) co-occurring habitat and geographic location entities within every sentence in the corpus. Two annotators manually provided the label *y* for each data instance (*I, E*_*S*_, *E*_*T*_, *y*). Our senior annotator, a Biology degree holder, labeled all instances in the entire dataset, while the junior annotator, a Computer Science student, provided labels for only 30% of the same dataset. Our annotators manually determined whether a pair of co-occurring entities are semantically related to each other (*y* = 1). We calculated the agreement between our two annotators in terms of F1-score, and obtained an overall agreement of 95.87%. Specifically, the agreement for the has_time relation type is 94.36%, while that for the has_location type is 97.37%. We resolved the disagreements by involving a third annotator who is a co-author of this paper. The instances with disagreements were re-evaluated and re-labeled by the third annotator. We randomly split our dataset into proportions of 70, 10, and 20% to serve as our training, development, and test sets, respectively. Table 7 shows the number of instances for each relation type.

**Table 7:** Frequency of instances for each relation type in our training (train), development (dev) and test sets.

**Relation type**	**Train**	**Dev**	**Test**
has_time	843	173	388
has_location	252	34	127

### 6.2 Relation extraction

In this section, we report the results of our experiments for evaluating performance of the unsupervised approaches we developed to extract has_time and has_location relations. We designed rules and templates based on our training set. The rules were then refined based on our held-out development (dev) set. Finally, the performance of the approaches was evaluated using the test set. We present the performance of each of our rule-based, transformer-based, and hybrid RE approaches in terms of precision, recall, F1-score, and Matthews correlation coefficient (MCC). Precision tells us how much we can trust the model when it predicts an example as positive, while recall measures the ability of the model to find all the positive examples in the dataset. The harmonic mean of precision and recall is the F1-score, which is useful in finding the best trade-off between the two values (Grandini et al., 2020). While the F1-score is widely used and is considered to be the standard performance metric for RE, MCC offers a more balanced assessment by considering all aspects of the confusion matrix, i.e., the number of true positives, false positives, false negatives, and true negatives (Chicco and Jurman, 2020). The range of MCC is [−1, 1]. It produces a high score only if good results were obtained for all of the four confusion matrix categories, proportional to the number of positive examples and the number of negative examples in the dataset, making it suitable for imbalanced datasets (Chicco and Jurman, 2020, 2023).

#### 6.2.1 Rule-based approaches

As explained in the Methods section (Section 5.1), we designed two rule-based approaches for RE, i.e., patterns based on dependency distance and regular expression-based rules (regexes). The patterns based on dependency distances were implemented using the command line interface of the *Grew-match* package (version 1.11). Using these patterns, we extracted related biodiversity entities whose dependency distance is at most *n*, where 1 ≤ *n* ≤ 8. For example, if *n* is set to 5, we extract relations between entities that have a dependency distance of 5 or less. It is worth noting that as the dependency distance increases, the extractions obtained by the dependency distance-based patterns become similar to those of a simple co-occurrence-based method that considers every pair of entities as related as long as they appear within the same sentence. For the purposes of our evaluation, we chose 4 as the value of *n*, as it yielded the highest F1-score for the has_location relation type on the development set. As described in Section 5.1, we also developed regular expression-based rules to extract related consecutive entities. The *re* Python module was used in implementing these regex-based rules.

Table 8 shows the precision, recall, F1-score and MCC of our two rule-based approaches, compared with a simple co-occurrence-based method as a baseline. The patterns based on dependency distance obtained F1-scores of 78.02 and 85.58% for the has_location and has_time relations, respectively, based on evaluation on our test set. These are lower than the F1-scores of a simple co-occurrence-based approach, which are 84.02% for the has_location relation, and 94.57% for the has_time relation. However, it should be noted that the dependency distance-based method has a higher precision than the co-occurrence-based one; the recall of the latter is 100% only because it classifies all instances as positive relations and none as negative.

**Table 8:** Precision (P), Recall (R), F1-score (F), and Matthews correlation coefficient (MCC) values obtained by rule-based approaches on the test set for has_time and has_location relation types.

**Rule-based methods**	has_time				has_location			
	**P(%)**	**R(%)**	**F(%)**	**MCC**	**P(%)**	**R(%)**	**F(%)**	**MCC**
Co-occurrence	89.69	100.00	94.57	0.00	72.44	100.00	84.02	0.00
Dependency distance (*n* = 4)	94.14	78.45	85.58	0.25	78.89	77.17	78.02	0.23
Regular expression rules	100.00	33.91	50.64	0.22	100.00	36.96	53.97	0.37

Among our rule-based methods, the regular expression-based rules obtained the better MCC for the has_location relation type, with an MCC value of 0.37. Furthermore, they obtained perfect precision (100%) for both relation types. This means that every relation predicted as a positive example by our regex-based rules is indeed a correct relation. However, such rules obtained poor recall, i.e., 33.91 and 36.96% for the has_time and has_location relations, respectively, implying that the regexes fail to extract majority of correct relations described in text; this resulted in the lowest F1-scores among the methods that we evaluated.

#### 6.2.2 Transformer-based approaches

We performed experiments using more recent and state-of-the-art transformer-based models. As discussed in Section 5.2, we cast our relation extraction problem as boolean QA and NLI tasks, for which we created question and hypothesis templates. We employed these methods by building upon (1) RoBERTa-BoolQ, a RoBERTa model that was fine-tuned on the BoolQ dataset, (2) T5-BoolQ, a T5 model that was also fine-tuned on BoolQ, and (3) T5-Large NLI, a T5-large model that was already fine-tuned for the NLI task. All of these models are available in the Hugging Face platform. We used Google Colaboratory to apply these models to the RE problem in a zero-shot manner, i.e., without any fine-tuning on domain-specific data.

Table 9 shows the performance of the above-mentioned boolean QA and NLI models on RE. Applying the models and our templates on our test set gave us F1-scores that are higher than our dependency distance-based patterns and rule-based approaches. The F1-scores obtained by Roberta-boolQ and T5-boolQ are similar to, if not better than, the F1-scores of the co-occurrence-based method, with a difference of only < 1 percentage point. For the has_location relation type, the T5-NLI model produced the highest F1-score, 84.75%, while for has_time, T5-NLI yielded the lowest F1-score, 86.98%, among the three transformer models. However, this model obtained the highest precision and MCC among the three models, i.e., F1-scores of 97.16% and 88.24%, and MCC values of 0.4 and 0.5 for the has_time and has_location relation types, respectively.

**Table 9:** Precision (P), Recall (R), F1-score (F), and Matthews correlation coefficient (MCC) values obtained by transformer-based approaches on the test set for has_time and has_location relation types.

**Transformer models**	has_time				has_location			
	**P(%)**	**R(%)**	**F(%)**	**MCC**	**P(%)**	**R(%)**	**F(%)**	**MCC**
Roberta-BoolQ	91.94	98.28	95.00	0.36	72.44	100.00	84.02	0.00
T5-BoolQ	93.04	95.98	94.48	0.39	72.58	97.83	83.33	0.02
T5-Large NLI	97.16	78.74	86.98	0.40	88.24	81.52	84.75	0.50

#### 6.2.3 Hybrid approach: rules and transformers

In this section, we present the evaluation results for our hybrid approach, a two-step method comprised of our regular expression-based rules and transformer-based approaches (Rules + Transformer). The precision, recall, F1-scores, and MCC values of these methods are summarized in Table 10. This approach resulted in an increase in the MCC value of up to 0.06 for all the models. Additionally, it produced a slight improvement on the F1-scores of Roberta-boolQ and T5-boolQ. In the case of T5-NLI, the initial step of rule-based analysis improved the F1-score for has_location relation extraction from 84.75 to 85.39%, and from 86.98 to 89.61% for the has_time relation type. Apart from improved performance, our hybrid approach is also more efficient, in that it requires the application of the more computationally expensive transformer models only on instances that were not classified by the rule-based approach as pertaining to relations.

**Table 10:** Precision (P), Recall (R), F1-score (F), and Matthews correlation coefficient (MCC) values obtained by hybrid approaches on the test set for has_time and has_location relation types.

**Hybrid approaches**	**Transformer models**	has_time				has_location			
		**P(%)**	**R(%)**	**F(%)**	**MCC**	**P(%)**	**R(%)**	**F(%)**	**MCC**
Rules, Transformer	Roberta-BoolQ	92.00	99.14	95.44	0.41	72.44	100.00	84.02	0.00
	T5-BoolQ	93.15	97.70	95.37	0.45	72.58	97.83	83.33	0.02
	T5-Large NLI	97.31	83.05	89.61	0.45	88.37	82.61	85.39	0.52
Rules, Compound, Transformer	Roberta-BoolQ	91.80	99.71	95.59	0.43	72.44	100.00	84.02	0.00
	T5-BoolQ	91.40	97.70	94.44	0.27	72.36	96.74	82.79	-0.01
	T5-Large NLI	95.26	98.28	**96.75**	**0.64**	83.96	96.74	**89.90**	**0.58**

The best F1-scores and MCC values are shown in bold.

We further improved our hybrid approach by using compound entities identified using regex rules in generating inputs to the transformer models (Rules + Compound entities + Transformers), instead of separate single entities. Evaluating this approach on our test set, we determined that the T5-NLI model produced the highest F1-scores and MCC values among all the methods that we experimented with. The computed F1-score is 89.90% for the has_location relation type, and 96.75% for the has_time relation type, while the MCC is 0.58 and 0.64 for the has_location and has_time relation types, respectively.

## 7 Discussion

In this section, we analyze the performance of our unsupervised RE approaches for biodiversity entities. This is followed by a discussion of the quality of the annotations in our corpus, an overview of the implications of our results, as well as the limitations of our work.

### 7.1 Comparative analysis

Our regex-based approach is the most precise among all the approaches we developed in this study. However, it is also the approach that obtained the lowest recall. It misses to identify more than half of true relations in the test set. The regex-based approach is suitable for applications that cannot compromise high levels of precision, e.g., systems that support clinical decisions, or the automatic curation of databases. The main drawback of the regex-based approach is its dependency on syntactic similarity only, i.e., solely on similar word order patterns found within sentences.

The other rule-based approach we developed uses the dependency distance between entities as basis for relation extraction. This approach relies on the selection of a value for maximum dependency distance *n*, which determines the trade-off between higher recall and poorer precision. That is, a low value for *n* provides us with high precision. As we increase the value of *n*, recall increases as well. However, precision decreases and approaches the performance of a simple co-occurrence-based RE method. Among our approaches, we found that the value of *n* was most difficult to fine-tune.

The drawback of our rule-based approaches is their reliance on syntactic structure only. Both our rule-based approaches (i.e., the regexes and the patterns based on dependency distance) are quite sensitive to noisy data and do not consider any semantics. Any deviation from typical sentence structures would affect the performance of the rule-based RE method.

Among the approaches presented in this paper, our transformer-based approaches were most straightforward to implement. The formulation of question templates for the boolean QA models, and hypothesis templates for the NLI model are based on natural language and do not require know-how of the English grammar nor of programming. The transformer models paired with our question/hypothesis templates for RE provided us with F1-scores higher than our rule-based methods. For the has_time relations, the QA models produced high F1-scores. However, the NLI model obtained the highest precision, 97.16%, and highest MCC, 0.45. For the has_location relations, the NLI model is the most precise and has the highest F1-score. For both relation types, the NLI model obtained the lowest recall.

During method development and preliminary evaluation on our development set, we noticed the high precision of the regex-based approach and the transformer-based model's higher recall, compared to that of the regexes. This led us to the idea of combining the high-precision regex-based approach and the high-recall transformer-based approach. Thus, we developed a hybrid approach that is a two-step method comprised of the regex-based method followed by a transformer-based one. This hybrid approach increased the recall for has_time relations by up to 4.31 percentage points, and the recall for has_location relations by 1 percentage point. We inspected some instances which the hybrid approach failed to identify as a relation. We noticed that this approach failed to identify relations between entities that belong to an enumeration or a compound statement of entity mentions. For example, in the sentence “*Ashton (**1989**) record the extent of mass flowerings in peninsular Malaysia and Borneo for the period 1950–1983 based on state forest department records (**Table 5**),”*^8^ the hybrid approach failed to determine that there is a relationship between “*mass flowerings”* and “*1983.”* Thus, as an enhancement to the hybrid method, we created regex-based rules to identify compound entities in sentences, as described in Section 5.3. Where they exist, these compound entities were used in populating the question and hypothesis templates, instead of individual named entities. The inclusion of regexes for compound entities in our hybrid approach significantly improved the recall of the NLI model by 14–15% points. This hybrid approach underpinned by the NLI model provided us with the highest F1-scores and MCC values among all the approaches we developed for both relation types.

### 7.2 Quality of annotations

Our results show that the annotations in our labeled corpus are reliable, given that a high level of agreement between the two annotators was obtained. Most of the disagreements were due to human errors that can be expected, e.g., missed relations when at least one of the entities belongs to an enumeration, or wrong interpretation of a complex sentence. Aside from these errors, there were very few instances of disagreements that were due to difficult cases that required deep knowledge of domain-specific terminology, e.g., when our junior annotator failed to determine that there is a semantic relationship between “*GF”* and “*February 2002”* in the sentence “*We quantified pre-dispersal seed predation for focal trees for all species of the genus Shorea section mutica having five or more qualifying individuals in February 2002, September 2002 and August 2005 (for the 2001, 2002, and 2005 GF events, respectively).”* Our more senior annotator (who has a Biology background) is more knowledgeable in reproductive conditions and was able to determine that, e.g., “*GF”* is an abbreviation for *general flowering*, and that “*dispersal”* implies the existence of fruit.

### 7.3 Theoretical and practical implications

From a theoretical perspective, our work in developing unsupervised RE methods for the biodiversity domain advances the NLP task of RE by proposing traditional rule-based, transformer-based, and a hybrid of rules and transformer models that extract related biodiversity entities without requiring a large amount of labeled training data. Our work has led to the development of unsupervised approaches for RE that can perform at a satisfactory level, according to the results of our evaluation. Our main methodological contribution to the NLP research community is the development of a two-step hybrid approach employing a high-precision regex-based method followed by a high-recall transformer-based NLI model, that obtains superior performance on the RE task.

From a practical perspective, our research has several applications. Firstly, our solution has the potential to support the curation of structured biodiversity data resources (e.g., databases, knowledge graphs) with finer-grained information on species' reproductive conditions and habitats. For instance, by extracting related mentions of reproductive conditions and temporal expressions from text, our method facilitates the inclusion of temporally specific reproductive conditions into a database or knowledge graph. This also applies to our other relation type of interest (i.e., the has_location relation type) that extracts geographically defined habitat information from text. Our approach can thus be applied to the enrichment of information in well-known biodiversity databases such as the Global Biodiversity Information Facility (GBIF) and the Atlas of Living Australia (ALA).

Furthermore, as part of a user-facing application, our RE approach can facilitate the extraction of detailed information (e.g., geographically broad-scale and long-term information on species' reproduction and habitats) from unstructured data, that can inform the decision-making of natural resource management (NRM) regulators. This can aid the implementation of data-driven approaches to land restoration and rehabilitation, which is part of the United Nations Sustainable Development Goal #15: Life on Land. Extracting has_time and has_location relations can help researchers in understanding the biology underpinning the proper timing for seed collection and seeding at suitable locations, which are important factors for effective regeneration and reforestation efforts. Once the extracted information has been represented in a structured form such as a knowledge graph, the entities and the relations between them can be easily visualized. This provides a more practical and intuitive way of viewing and analyzing reproductive condition—temporal expression and habitat—geographic location relations, compared to reading lengthy textual documents.

### 7.4 Limitations

To the best of our knowledge, the biodiversity-focused corpus proposed by Gabud et al. (2019) is the only publicly available corpus containing labels pertaining to reproductive condition, temporal expression, habitat and geographic location named entities. Hence, the methods we developed for extracting reproductive condition - temporal expression (has_time) and habitat - geographic location (has_location) relations were based on this corpus only. In this work, we show that despite the relatively small size of the RE-annotated corpus resulting from our work, we were able to develop unsupervised approaches for RE that perform at a satisfactory level. However, this corpus might be too small to support the training of traditional supervised machine learning-based RE models.

## 8 Conclusions and future Work

In this paper, we present our unsupervised relation extraction methods to extract relationships pertaining to habitats and reproductive conditions of plant species in text. We present our relation extraction corpus that contains manually labeled relations between mentions of reproductive conditions and temporal expressions (i.e., has_time relations), and between habitats and geographic locations (i.e., has_location relations). We used this corpus to experiment with our three unsupervised methods for relation extraction, namely, a rule-based approach, a transformer-based one, and a hybrid approach that combines the first two. Our rule-based approaches are based on patterns in the dependency distance between entities, and regular expressions that capture the word order of entities. They obtained the highest precision but were poor in terms of recall. For the transformer-based approach, we framed our relation extraction problem as boolean QA and NLI tasks. The methods we experimented with using this approach resulted in F1-scores higher than those obtained by the rule-based approach. We designed our two-step hybrid approach by combining our rules with our transformers models. A further improvement is the addition of using compound entities in generating the question (for boolean QA) or hypothesis (for NLI) instead of single entity mentions. Our hybrid approach composed of rules, compound entities, and a transformer-based NLI model (built upon T5-large) produced the best performance, with a 96.75% F1-score for related reproductive conditions and temporal expressions, and a 89.90% F1-score for related habitats and geographic locations. Our work shows that even without a large training dataset, we have been able to extract has_location, and has_time relations from literature with satisfactory performance.

We consider our work to be a contribution toward large-scale studies on biodiversity that impacts sustainable life on land. This could eventually facilitate the development of a biodiversity database enriched with information on the habitats and reproductive conditions of species, extracted from literature. As part of our future work, we plan to analyze the extent to which our pre-processing steps (e.g., tokenization, dependency parsing) affects RE performance. Furthermore, we intend to implement an information extraction pipeline comprised of an NER tool and our hybrid RE approach that automatically identifies related biodiversity entities from text. We will explore how to incorporate such reproductive condition and habitat information from text into species occurrence data stored in databases such as GBIF and ALA.

## Data availability statement

The raw data supporting the conclusions of this article will be made available by the authors, without undue reservation.

## Author contributions

RG: Formal analysis, Investigation, Methodology, Software, Writing - original draft, Writing - review & editing. PL: Writing - review & editing, Data curation. VM: Formal analysis, Writing - review & editing. EM: Formal analysis, Writing - review & editing. NP: Writing - review & editing, Data curation. MC: Formal analysis, Writing - review & editing. RB-N: Conceptualization, Writing - original draft, Writing - review & editing, Formal analysis.

## Footnotes

^1^Available at: https://nlp.stanford.edu/software/stanford-dependencies.html.

^2^ https://universaldependencies.org/format.html

^3^ https://grew.fr/

^4^ https://huggingface.co/

^5^Available at: https://huggingface.co/shahrukhx01/roberta-base-boolq.

^6^Available at: https://huggingface.co/mrm8488/t5-base-finetuned-boolq.

^7^Available at: https://huggingface.co/t5-large.

^8^Source: Appanah (1993).
